# The Practice of Surgery

**Published:** 1846-06

**Authors:** 


Art. X.—*The Practice of Surgery.* By James Miller, F.R.S.E., F.R.C.S.E., Professor of Surgery in the University of Edinburgh; Surgeon to the Royal Infirmary; Author of Principles of Surgery, &c., &c. Philadelphia: Lea & Blanchard. 1846. 8vo. pp. 496.

So numerous of late have been the publications on Surgery in this country, that another volume on the same subject would hardly seem to be called for. The Lectures of Colles and Brodie, the popular volumes of Liston, and the works of the famous Sir A. Cooper, have all been issued within a few years, not to speak of the Principles of Surgery by the author of the present work, the reprint of Cooper's Surgical Dictionary, the New York edition of Velpeau's elaborate treatise on Surgery, and the splendid quarto volume on Operative Surgery by Dr. Pancoast. Judging from the quick succession in which these works have followed each other, the surgical spirit of our country must be high. The author of the volume before us modestly disclaims any intention to put it forth "in rivalry of the excellent works on practical surgery which already exist." It is intended as a companion to his "Principles of Surgery," the two volumes together forming a text-book, which he undertook at the request of his pupils. The fact that they have been adopted by other teachers of surgery, as text-books, is decisive proof of their adaptation to the wants of the learner. The manner of the author is the best, being simple, concise, and clear. The practitioner who has occasion to look into its pages, will find the *practice of Surgery* laid down in a readable compass, without circumlocution, or unnecessary citation of authorities.

